# Mineral and Amino Acid Profiling of Different Hematopoietic Populations from the Mouse Bone Marrow

**DOI:** 10.3390/ijms21176444

**Published:** 2020-09-03

**Authors:** Mukul Girotra, Caroline Monnard, Tobias Konz, Federico Sizzano, Laurence Goulet, Jean-Philippe Godin, George Coukos, Serge Rezzi, Nicola Vannini

**Affiliations:** 1 Department of Oncology, Ludwig Cancer Institute, University of Lausanne, 1066 Epalinges, Switzerland; mukul.girotra@unil.ch (M.G.); George.Coukos@chuv.ch (G.C.); 2 Nestlé Research, EPFL Innovation Park, 1015 Lausanne, Switzerland; monnard.caroline@gmail.com (C.M.); tobias.konz@gmail.com (T.K.); Federico.Sizzano@rd.nestle.com (F.S.); Laurence.Goulet@rd.nestle.com (L.G.); serge.rezzi@swissvitamin.ch (S.R.); 3 Nestlé Research, Vers-chez-les-Blanc, 1000 Lausanne, Switzerland; jean-philippe.godin@rdls.nestle.com

**Keywords:** hematopoietic stem and progenitor cells, amino acids, minerals, mitochondria, urea cycle

## Abstract

Steady hematopoiesis is essential for lifelong production of all mature blood cells. Hematopoietic stem and progenitor cells (HSPCs) found in the bone marrow ensure hematopoietic homeostasis in an organism. Failure of this complex process, which involves a fine balance of self-renewal and differentiation fates, often result in severe hematological conditions such as leukemia and lymphoma. Several molecular and metabolic programs, internal or in close interaction with the bone marrow niche, have been identified as important regulators of HSPC function. More recently, nutrient sensing pathways have emerged as important modulators of HSC homing, dormancy, and function in the bone marrow. Here we describe a method for reliable measurement of various amino acids and minerals in different rare bone marrow (BM) populations, namely HSPCs. We found that the amino acid profile of the most primitive hematopoietic compartments (KLS) did not differ significantly from the one of their direct progenies (common myeloid progenitor CMP), while granulocyte-monocyte progenitors (GMPs), on the opposite of megakaryocyte-erythroid progenitors (MEPs), have higher content of the majority of amino acids analyzed. Additionally, we identified intermediates of the urea cycle to be differentially expressed in the KLS population and were found to lower mitochondrial membrane potential, an established readout on self-renewal capability. Moreover, we were able to profile for the first time 12 different minerals and detect differences in elemental contents between different HSPC compartments. Importantly, essential dietary trace elements, such as iron and molybdenum, were found to be enriched in granulocyte-monocyte progenitors (GMPs). We envision this amino acid and mineral profiling will allow identification of novel metabolic and nutrient sensing pathways important in HSPC fate regulation.

## 1. Introduction

The hematopoietic system generates billions of blood cells every day during an organism’s lifetime [1]. This constant cell turnover is maintained by a small pool of hematopoietic stem and progenitor cells (HSPCs) residing in the complex bone marrow (BM) microenvironment, called the niche [2]. A fine balance of self-renewal and differentiation fate choices allows the hematopoietic stem cells (HSCs) to maintain their own population and produce lineage restricted progenitors to fulfil the demands of the blood system. Remarkably, metabolism and dietary intervention have emerged as key contributors to this highly regulated process, which is controlled by both cell intrinsic, as well as extrinsic cues. Adult HSCs are mostly quiescent [3], and therefore have lower energy requirements. They were shown to rely predominantly on glycolysis [4] and fatty acid oxidation (FAO) [5], as opposed to downstream progenitors that fulfil their energy requirements by elevated mitochondrial oxidative phosphorylation (Oxphos) [6,7]. Interestingly, inhibition of Cpt1, the rate limiting enzyme of FAO, was shown to reduce leukemic proliferation [8].

Although lower protein translation rates in adult HSCs [9,10] were linked to their predominant state of quiescence when compared to rapidly dividing progenitors, several amino acids emerged as key regulators of HSC function. Recently, valine was shown to be critical for ex vivo mouse and human HSC proliferation and for retention in the BM [11]. Moreover, glutamine uptake and metabolism was found to be critical for HSPC differentiation into erythroid lineage and important for de novo nucleotide synthesis [12]. In contrast, glutamine starvation lead to inhibition of certain myeloid leukemias [13]. The availability of leucine, an essential amino acid, is believed to regulate nutrient sensing pathways, such as the mTOR pathway, in HSCs [14]. Other branch chain amino acids such as isoleucine and valine also contribute to cellular nitrogen balance and mTOR activity [15]. Moreover, nutrient availability regulates the integrated stress response (ISR) pathways which are believed to promote the survival of healthy HSPCs [16].

Other essential nutrients such as vitamins have also been implicated in the maintenance of the hematopoietic system. Vitamin A (and retinoic acid signaling) was shown to maintain HSC dormancy, while its absence in the diet lead to HSC impairment, possibly by uncontrolled differentiation [17]. Vitamin D was shown to be important for HSC self-renewal in the recently characterized Zebrafish hematopoietic system and was further extended to human cord blood derived HSPCs [18]. Additionally, a recent study reported the role of Vitamin D receptor expression in myeloid progenitor differentiation and also regulating hematopoietic and leukemic stem cell numbers [19]. Interestingly, mouse HSCs were found to be enriched in Vitamin C (ascorbate) as opposed to all downstream hematopoietic progenitors, and genetic deletion of ascorbate synthesizing enzyme (Gulo) resulted in increased HSC frequency and reconstitution capability [20]. More recently, nicotinamide riboside (NR), an analog of vitamin B3, has been demonstrated to be able to maintain HSC functions and promote self-renewing cell division [21,22].

External nutrition was also shown to influence hematopoietic function. Fasting could improve HSC function via inhibition of IGF1 signaling [23] while a high fat diet reduced HSC function [24,25,26], probably via the expansion of adipocytic cell populations in the bone marrow [27,28]. In mouse models of diabetes, HSCs were found to have poor mobilization and were mostly blocked in the bone marrow [29]. In addition to external sources, HSPCs are believed to recycle their internal cellular material and nutrients by maintaining high autophagy and mitophagy levels [30].

All these evidences highlight the importance of specific nutrients in maintaining HSC pool and preserving their function. In this study we characterize the intracellular amino acid and mineral contents of different hematopoietic stem and progenitor compartments by applying state-of-the-art mass spectrometry approaches [31]. Nutrient profiling of HSPCs revealed important differences between progenitor compartments, indicating that different hematopoietic stem and progenitor populations have specific nutrient requirements which could play a role on HSPCs fate decision and commitment.

## 2. Results and Discussion

### 2.1. Estimation of Amino Acid Content in Various Bone Marrow Populations

**Figure 1:**
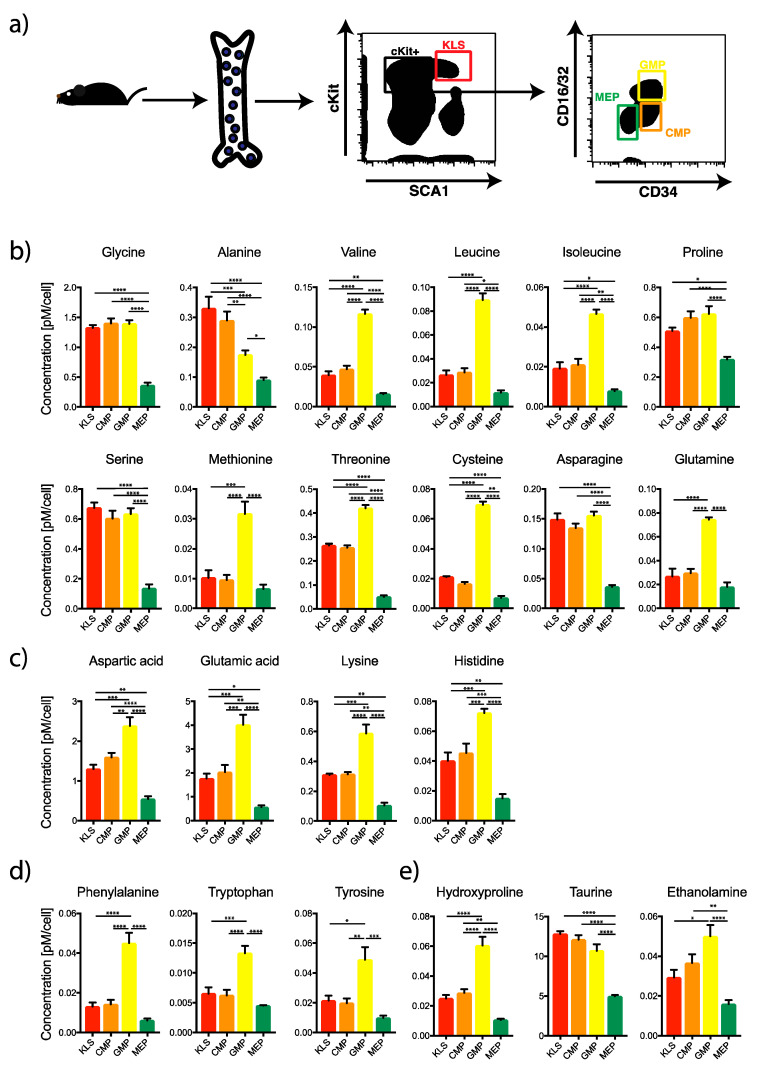
Estimation of amino acid content in various bone marrow populations. (**a**) Different populations from the mouse BM were FACS sorted for amino acid analysis. KLS: Lin^−1^cKit^+^Sca1^+^; CMP: Common Myeloid Progenitor; GMP: Granulocyte Macrophage Progenitor; MEP: Megakaryocyte Erythroid Progenitor. (**b**–**e**) Measurement of amino acid content in different populations. One-way ANOVA * *p* < 0.05, ** *p* < 0.01, *** *p* < 0.001, **** *p* < 0.0001 (*n* = 3).

We first purified the hematopoietic stem and progenitor cell (HSPC) populations from mouse bone marrow by FACS sorting. HSPCs were identified in the lineage negative fraction of the bone marrow as follows: KLS (Lin^−1^cKit^+^Sca1^+^) representing the multipotent stem and progenitor cells; CMP: common myeloid progenitor (Lin^−1^cKit^+^Sca1^−1^CD16/32^−1^CD34^+^); GMP: granulocyte macrophage progenitor (Lin^−1^cKit^+^Sca1^−1^CD16/32^+^CD34^+^); and MEP: megakaryocyte erythroid progenitor (Lin^−1^cKit^+^Sca1^−1^CD16/32^−1^CD34^−1^) (Figure 1a and Supplementary Figure S1).

**Figure 2:**
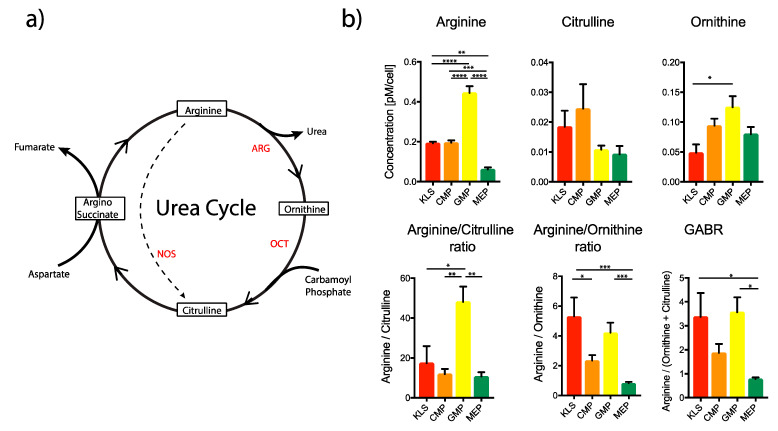
Estimation of Urea cycle intermediates in various bone marrow populations. (**a**) Schematic of the urea cycle. ARG: Arginase; OCT: Ornithine Carbamoyltransferase NOS: Nitric Oxide Synthase. (**b**) Urea cycle intermediates were measured in different populations from the mouse BM. KLS: Lin^−1^cKit^+^Sca1^+^; CMP: Common Myeloid Progenitor; GMP: Granulocyte Macrophage Progenitor; MEP: Megakaryocyte Erythroid Progenitor. One-way Anova * *p* < 0.05, ** *p* < 0.01, *** *p* < 0.001, **** *p* < 0.0001 (*n* = 3).

We then analyzed the cellular concentration of 25 different amino acids in HSPC populations using liquid chromatography-tandem mass spectrometry (LC-MS/MS) (Figure 1b–e and Figure 2b). In general, GMP and MEP, two populations derived from a common progenitor, namely CMP [32] had very different amino acid profiles. (Figure 1b–e). For most amino acids, the concentration was significantly higher in GMPs. Elevated amino acid content in GMPs reflects their faster proliferation rate as compared to other HSPCs [33], requiring substantial amounts of amino acids to gain cellular mass for cell-division [34].

Moreover, in GMPs, enrichment of branched amino acids, valine, leucine, and isoleucine (Figure 1b) could promote their transamination and degradation (by branched-chain alpha-keto acid dehydrogenase complex (BCKDH)) leading to acetyl-CoA generation, which, in turn, would fuel the TCA cycle in the mitochondria [35]. Elevated TCA cycle activity not only sustains mitochondrial respiration, but can also provide important substrates necessary for the production of biomolecules and epigenetic modifications [36]. In addition, all essential amino acids (Val, Leu, Ile, Met, Thr, Lys, His, Phe, and Trp) were enriched in GMPs (Figure 1b–d) while several non-essential amino acids, such as Gly and Ser, were equally enriched in the KLS and progenitor (CMP and GMP) population (Figure 1b), suggesting a yet unexplored biological significance of this segregation. Moreover, when we compared the intracellular content of Gly and Ser in HSPCs to other terminally differentiated blood cells, like CD8^+^ naïve T cells, we found glycine to be unchanged but, surprisingly, we found serine to be highly enriched in HSPCs as compared to CD8^+^ naïve T cells (Supplementary Figure S2). Serine has been recently reported as a key metabolite promoting T cell expansion via boosting one-carbon-metabolism [37], and our data indicate that possibly one-carbon-metabolism could have a role on HSPCs function and commitment by promoting nucleotide biosynthesis and chromatin remodeling. Alanine, another non-essential amino acid, was found to be significantly enriched in the KLS and CMP cells as compared to all other progenitor populations and demonstrated a systematic step-wise decrease along the hematopoietic hierarchy (Figure 1b). Interestingly we found that the alanine content in CD8^+^ naïve T cells was similar to KLS and CMP (Supplementary Figure S2). Interestingly, enrichment of alanine and low levels of glutamate in KLS could indicate an upregulation of the alanine cycle, where pyruvate and glutamate are converted in alanine and α–ketoglutarate by the enzyme alanine transaminase (ALT). Indeed, stem and progenitor cells have been shown to have low mitochondrial activity [6,7] and reduced uptake of pyruvate in the TCA cycle by upregulation of the enzyme PDK2 which governs the conversion of pyruvate into acetyl-CoA [4,22]. Therefore, the upregulation of the alanine cycle could be an additional mechanism aimed to reduce acetyl-CoA uptake into the mitochondria. Congruently, SLC1A5/ASCT2, the alanine, serine, and cysteine transporter 2 is enriched in HSPCs; however, the lack of SLC1A5 results in minor defects in blood development in steady state conditions, while it decreases leukemic initiation in genetic mouse model [38].

The amino acid taurine, an important regulator of cellular redox homeostasis [39], was also found in higher levels (although significant only with MEP) in the more primitive populations (KLS and CMP) (Figure 1e). Taurine functions as free radical scavenger [40,41] and its depletion has been shown to increase mitochondrial free radicals [42]. Importantly, HSCs maintain low levels of ROS to limit oxidative damage and maintain stable hematopoiesis throughout an organism’s lifetime [43]. In this context, taurine could synergize with other antioxidant pathways [44,45,46,47] to limit ROS-induced damages in the most primitive hematopoietic compartments.

We also analyzed arginine metabolism and other intermediates of the urea cycle (Figure 2a), such as citrulline and ornithine, to ascertain their possible functional relevance in different HSPC populations, being important modulators of immune function [48,49]. Arginine was enriched in the GMP population (Figure 2b), similar to the pattern observed for essential amino acids. However, we found citrulline to tend to be enriched in KLS and CMP as compared to GMP (Figure 2b) while ornithine, another important intermediate in the urea cycle, had the opposite trend (Figure 2b).

Ornithine is derived from the direct conversion of arginine by the enzyme arginase (ARG), while citrulline can be derived both from the direct conversion of arginine, by the enzyme nitric oxide synthase (NOS), or from the conversion of ornithine, by the enzyme ornithine carbamoyltransferase (OCT) [50]. Although arginine and ornithine were not enriched individually in the KLS population, we found the arginine/ornithine ratio, a value inversely corelated to arginase activity [51], to be the highest in KLS cells, indicating a reduced arginase activity and urea production (Figure 2b). On the other hand, the arginine/citrulline ratio was found to be significantly lower in the KLS than GMPs, indicating an increased NOS activity and NO production (Figure 2b). This might partially explain the enrichment of taurine, a potent scavenger of NO [41], in the KLS population as previously discussed (Figure 1e). Furthermore, we found the global arginine bioavailability ratio (GABR), defined as the ratio between arginine and its major metabolites (ornithine and citrulline), and reported as index of arginine bioavailability, to be higher in the KLS and GMP populations (Figure 2b).

### 2.2. Ex Vivo Culture of HSPCs with Urea Cycle Intermediates

**Figure 3:**
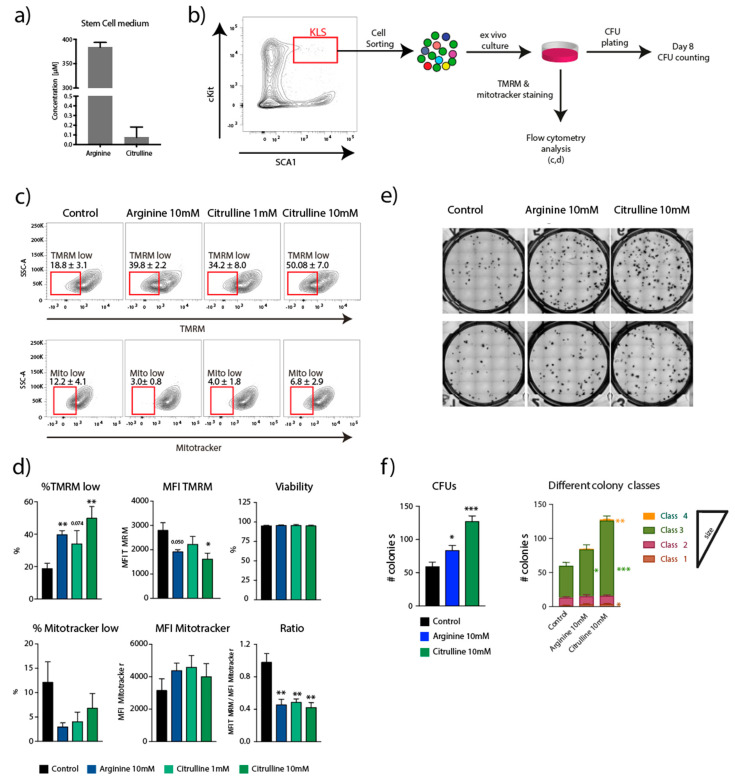
Ex vivo culture of HSPCs (KLS) with Urea cycle intermediates. (**a**) Measurement of arginine and citrulline concentration in stem cell media. (**b**) FACS sorting of KLS population for ex vivo culture in 96 well round bottom plates, followed my TMRM and Mitotracker staining for FACS analysis, and CFU plating. (**c**) Contour plots showing TMRM and Mitotracker levels in different conditions (**d**) Estimation of Viability of cells post culture, TMRM and Mitotracker low population and Mean Fluorescence intensity (MFI) of TMRM and Mitotracker. (**e**) Representative images of CFU wells at Day 8 from control, arginine (10 mM) and citrulline (10 mM) conditions. (**f**) Estimation of colony counts and different colony classes as identified by STEMvision analyzer software. Student *t*-test * *p* < 0.05, ** *p* < 0.01, *** *p* < 0.001, (*n* = 4).

Having identified urea cycle as an important pathway operating in the most primitive hematopoietic compartment, we tested urea cycle intermediates in ex vivo cultures and determined their impact on the metabolic profile of KLS cells. To this end, we used TMRM and mitotracker green staining (Figure 3b) as indirect readout on mitochondrial membrane potential (ΔΨm) and mass respectively [7,52]. We have previously shown that low TMRM levels (aka low ΔΨm) is a hallmark of self-renewing HSCs with improved in vivo blood reconstitution capability [7]. Moreover, we demonstrated that artificially lowering ΔΨm using external agents is a reliable strategy to maintain stem cell function ex vivo [7,21,22,52].

First, using LC-MS/MS, we measured the amount of arginine and citrulline in the stem cell media used in our standard ex vivo cultures and found the concentration of arginine and citrulline to be at ~375 µM and ~0.1 µM, respectively (Figure 3a). We sorted the KLS population using FACS and cultured them for three days at high concentrations of arginine and citrulline, 10 mM and 1–10 mM, respectively. At the end of the culture, the cells were stained with TMRM and Mitotracker green, allowing a readout both on ΔΨm and mitochondrial mass (Figure 3b). Both, arginine and citrulline, were able to expand the proportion of cells in the TMRM low gate (representative plots, Figure 3c), which correlated with a concomitant decrease of the mean fluorescence intensity (MFI) of both TMRM and mitotracker green (Figure 3d). Importantly, these data highlight the capacity of arginine and citrulline in reducing ΔΨm/mitochondrial activity, and thus, as we have previously demonstrated, possibly improving stem cell function [7,22]. Furthermore, we performed CFU assay of cells exposed to arginine and citrulline to access HSPC function (Figure 3e,f). Importantly, we confirmed, arginine and citrulline induced reduction in ΔΨm results in improvement in colony formation (Figure 3e,f). Moreover, we found a significant increase in class 3 and class 4 type of colonies (which are derived from most primitive HSPCs), in the treated conditions (Figure 3f).

Although, supplementation of arginine and citrulline did not show significant changes in mitochondrial mass (as measured by Mitotracker green) (Figure 3c,d), it showed a significant lowering of TMRM/Mitotracker ratio, indicating that the mitochondrial activity per mitochondria was decreased in the presence of arginine and citrulline (Figure 3d). To note, no toxic effect on KLS was found in response to the addition of these intermediates in the culture media (Figure 3d).

### 2.3. Estimation of Minerals and Trace Elements in Various Bone Marrow Populations

Minerals play important roles in several cellular processes, where they act as enzyme cofactors, as signal transduction molecules or electrolytes [31]. Measurement of minerals in different hematopoietic populations has been hampered by limitations due to the sample size requirements of the established methodologies. In this context, we have recently developed a robust and reliable method enabling the measurement of the complete panel of biological relevant minerals and trace elements in small size samples by inductively-coupled plasma mass spectrometry (ICP-MS) [31]. We then applied this methodology to measure elements in different mouse BM populations, previously defined (Figure 1a).

**Figure 4:**
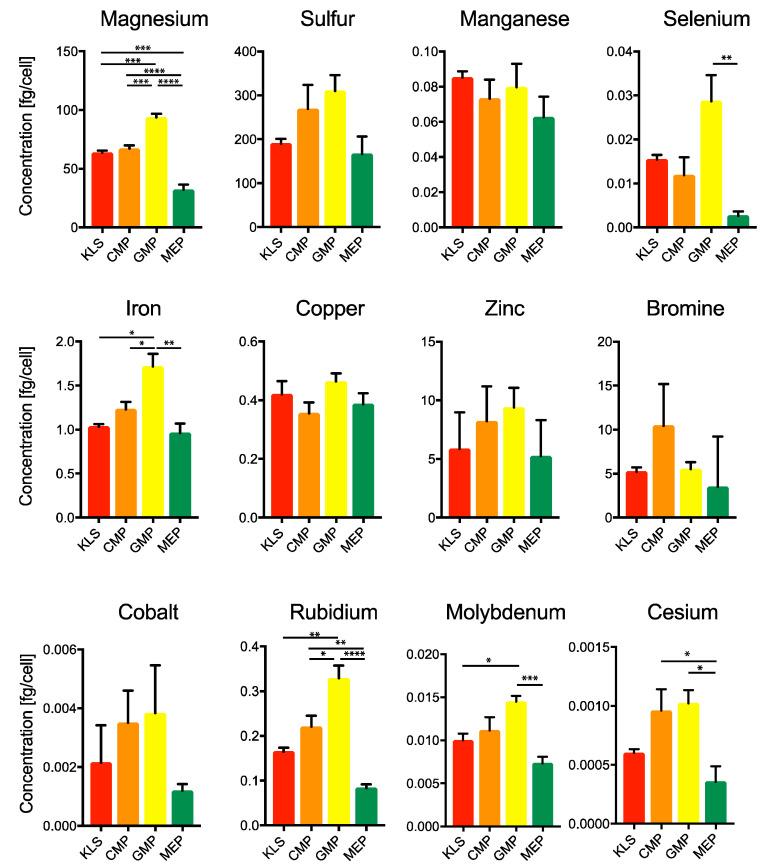
Estimation of minerals and trace elements in various bone marrow populations. Mineral analysis was performed on different bone marrow populations, as described in Figure 1. KLS: Lin^−1^cKit^+^Sca1^+^; CMP: Common Myeloid Progenitor; GMP: Granulocyte Macrophage Progenitor; MEP: Megakaryocyte Erythroid Progenitor. One-way ANOVA * *p* < 0.05, ** *p* < 0.01, *** *p* < 0.001, **** *p* < 0.0001 (*n* = 3).

Among all minerals, we found magnesium (Mg) significantly enriched in GMPs and depleted in MEPs as compared to KLS and CMPs, which display similar intracellular content (Figure 4). Given high energy requirements of the rapidly proliferating GMPs it is plausible that magnesium enrichment allows these cells to maintain a very high metabolic activity and elevated ATP production. Indeed, Mg is one of the most abundant cations in the cell and plays important roles in diverse cellular processes [53]. Mg is involved in the production and use of ATP, by forming ATP and ADP complexes, which function as substrates of key metabolic enzymes, or by direct binding to the glycolytic enzymes such as phosphofructokinase and pyruvate kinase, causing their allosteric activation [54,55].

Moreover, magnesium controls ATP production in the mitochondria by altering proton membrane permeability, therefore causing alterations in oxidative phosphorylation and electron transport chain [56,57,58]. A fine balance of cytosolic and mitochondrial magnesium regulates these processes under homeostasis [59]. Furthermore, by making complexes with plasma membrane phospholipids and ion channels it also regulates membrane permeability and ion movement [60]. For these reasons, magnesium is considered an extremely important regulator of cellular metabolic state. In future, it will be interesting to look at the ratio of cytoplasmic to mitochondrial magnesium concentrations to determine its effect on ATP production in these cells.

Selenium (Se), an important cofactor for the activity of peroxidase scavenger enzyme glutathione peroxidase [61,62], was also found to be highly enriched in the GMP population (Figure 4). On the other hand, manganese, a cofactor of another ROS scavenger enzyme super oxide dismutase (SOD), was equally present in all HSPC populations (Figure 4). These data indicate the possible importance of selenium and selenoproteins, such as glutathione peroxidase, in contributing to the RedOx balance of GMP population.

Moreover, iron (Fe) was enriched in GMPs, possibly localizing in the mitochondria and functioning as cofactor for complex I-IV of the respiratory chain, and thus contributing to the energy requirements of these rapidly dividing cells (Figure 4), at the same time Fe intracellular content was significantly lower in differentiated CD8^+^ naïve T cells (Supplementary Figure S2).

Molybdenum (Mo), an essential micronutrient was significantly elevated in the GMP population (Figure 4). Similarly, CD8^+^ naïve T cells had lower Mo intracellular content as compare to HSPCs (Supplementary Figure S2). Molybdenum, although catalytically inactive by itself, binds to pterin based molybdenum cofactor (Moco), present in the molybdenum enzymes, to catalyze several metabolic reactions in cells [63]. Interestingly, Moco is highly enriched in the immediate progeny of GMPs (Granulocytes and Monocytes) indicating a unique significance of molybdenum enzyme mediated processes in these cells. Additionally, we found cesium and rubidium to be enriched in CMPs and GMPs (Figure 4), however, to our knowledge their role in bone-marrow cells have not been described before and could be an interesting area to explore in the future.

In conclusion, we successfully measured amino acid and mineral content in different BM cell populations. Size analysis (using FSC) revealed a small (~10%) difference in size only between KLS and GMP (Supplementary Figure S3), this confirmed the pattern of enrichment in HSPCs is marginally influenced by size. We identified several amino acids differentially enriched in stem or progenitor populations, with arginine metabolism and urea cycle emerging as important pathways in stem cell regulation. Our TMRM assay revealed lowering of mitochondrial activity in multipotent stem and progenitor (KLS) population in response to supplementation of urea cycle intermediates in the culture media, suggesting a change in their fate. Elemental analysis showed magnesium (Mg), selenium (Se) and molybdenum (Mo) to be differentially enriched in various BM populations indicating their possible role in cellular processes and also regulating ROS scavenger enzymes. Moreover, comparison with terminally differentiated blood cells (CD8^+^ naïve T cells) indicated that many of these differences are characteristic of the HSPC populations from the BM (Supplementary Figure S2). However, due to technical limitation of starting material (high cells number requirement) for mass spectrometry-based analysis, to date, it is challenging to carry out amino acid profiling in highly purified HSCs and to our knowledge not possible for mineral content analysis. Therefore, in this study we restricted our analysis to KLS population which represents a mix population of stem and multipotent progenitor cells. Intriguingly, nutritional and metabolic interventions have also been suggested as treatments for disorders such as leukemia [64]. Elevated Vitamin C, known to support the absorption of non-heme iron in the gut, was shown to reduce leukemic progression while its depletion had the opposite effect [20]. Moreover, enzymes involved in the metabolism of branch chain amino acids were seen to be overexpressed in some acute and chronic myeloid leukemias [65,66]. We believe the analysis described in this study can be extended to BM populations from leukemic or aging patients in the future to identify possible nutritional deficits and develop nutritional intervention capable to restore HSPCs function.

## 3. Methods

### 3.1. Mice

C57Bl/6J female mice were purchased from Harlan (France) and were housed in pathogen-free facilities at University of Lausanne in micro-isolator cages. Mice were provided continuously with sterile food, water, and bedding. All experiments described in the manuscript follow the guidelines of our institution and were carried out in accordance with Swiss law for animal experimentation (authorization: VD3194).

### 3.2. Bone Marrow Extraction and Cell Sorting

Cell suspension was made by crushing femur, tibia, and pelvic bones of 8- to 10-week old female C57BL/6J mice in PBS-1mM EDTA. Cell suspensions were filtered through a 70 µm cell strainer and erythroid cells were eliminated by incubation with red blood cells lysis buffer (eBiosciences-Thermo Fisher Scientific, MA, USA). Lineage-positive cells were removed with a magnetic lineage depletion kit (Miltenyi Biotech, Germany). Cell suspensions were stained with a panel of specific antibodies for stem and progenitor cells and FACS-sorted on Beckman-Coulter Astrios. The following populations were identified, KLS: Lin^−1^cKit^+^Sca1^+^; CMP: common myeloid progenitor (Lin^−1^cKit^+^Sca1^−1^CD16/32^−1^CD34^+^); GMP: granulocyte macrophage progenitor (Lin^−1^cKit^+^Sca1^−1^CD16/32^+^CD34^+^); MEP: megakaryocyte erythroid progenitor (Lin^−1^cKit^+^Sca1^−1^CD16/32^−1^CD34^−1^). 200–250,000 sorted cells were collected in 1.5 mL Eppendorf tubes containing 100 μL PBS + 0.1% BSA.

### 3.3. Antibodies

Bone marrow cells were stained with the following antibodies cKit (2B8) PE/Cy7, Sca1 (D7) APC, CD34 (RAM) FITC, CD16/32 (93) AF700, Streptavidin- Pac Orange. A mixture of biotinylated mAbs against CD3, CD11b, CD45R/B220, Ly-6G, Ly-6C, and TER-119 was used as lineage cocktail (BD, NJ, USA). 7-AAD was used for live/dead cell discrimination

### 3.4. Ex Vivo Culture

Sorted KLS cells were culture for 3 days in 96-well round bottom well plate (4000 cells per well). Cultures were maintained in Stemline II (Sigma, MO, USA) supplemented with 100 ng/mL SCF (R&D Systems, MN, USA) and 2 ng/mL Flt3 (R&D), 50 U/mL Pen-Strep (Life technologies). Concentrations of arginine (SIGMA, MO, USA) and citrulline (SIGMA, MO, USA) were added as indicated in the main text.

### 3.5. TMRM and Mitotracker Staining

Post culture cells were incubated at 37 °C for 45 min with 200 nM TMRM (Invitrogen-Thermo Fisher Scientific, MA, USA) and 100 nM Mitotracker green. Cells were washed with FACS buffer and analyzed by flow cytometry on BD LSR II. The viability of cells was accessed using DAPI staining.

### 3.6. Amino Acid Measurement

The quantification of amino acids in cells (250,000 cells) was performed using an Acquity UPLC I Class system hyphenated to a TQS XEVO triple quadrupole from Waters (Milford, MA, USA). The following amino acids were measured: glycine, alanine, valine, leucine, isoleucine, proline, serine, methionine, threonine, cysteine, asparagine, glutamine, asprtatic acid, glutamic acid, lysine, histidine, phenylalanine, tryptophan, tyrosine, hydroxyproline, taurine, ethanolamine, arginine, citrulline, and ornithine. The chromatographic system was equipped with a binary pump, a temperature controlled autosampler operated at 20 °C and a column oven set to 55 °C. Chromatographic separation was achieved by a Waters Acquity AccQ Tag Ultra C18 column (1.7 µm, 100 × 2.1 mm I.D) and an Acquity UPLC BEH C18 VanGuard Pre-column (130 Å, 1.7 µm, 2.1 mm × 5 mm) applying a flow rate of 0.7 mL min^−1^. Mobile phase A consisted of 10% AccQ Tag Ultra Eluent A concentrate in H_2_O and AccQ Tag Ultra Eluent B (both from Waters) as mobile phase B. For sample injection, the full loop mode was applied to inject 10 μL of sample. The UHPLC system was hyphenated with the mass spectrometer employing electrospray ionization (ESI) in positive mode for ionization. Nitrogen was used as desolvation, nebulization and cone gas while argon was employed as collision gas. Collision energy was adapted to each analyte individually, ranging between 18 and 40 eV performed. Amino acids were identified according to the retention time and compound specific transitions. Quantitation was achieved using an internal standard and an external calibration curve.

The sample preparation was performed with the AccQ Tag kit from Waters (Milford, MA, USA). Briefly, calibration solutions were concentrated using a speed vac and 10 µL of each calibration solution was then mixed with 70 µL borate buffer (provided in the AccQ•Tag kit) before adding the derivatization agent. Samples were placed in a heating cabinet for incubation (55 °C, 10 min) and diluted with 0.1 M HCl prior analysis. For preparation of mouse samples, the cell pellets were mixed with internal standard solution and resuspended in ice cold acetonitrile, 0.1% formic acid (Sigma Aldrich, MO, USA). The mixture was homogenized by doing cycles of vortexing, sonication and incubation on dry ice. Samples were then centrifuged to remove precipitated proteins and concentrated using a speed vac. 10 µL of each sample was then mixed with borate buffer and derivatization agent, incubated at 55 °C for 10 min and diluted with 0.1 M HCl prior analysis.

The samples that had a signal/noise ratio of less than 10 or were below the limit of detection were excluded from the analysis. The concentration in amino acid or metabolite obtained was then normalized by dividing with the number of cells in the pellet (from the number of cells sorted).

### 3.7. Mineral Measurement

Mineral and trace element measurement was done using the protocol described by Konz et al. [31]. For each population and each repeat 250,000 cells were used for analysis.

### 3.8. CFU Assay

FACS sorted KLS cells were plated in different culture conditions in a 96 well plate (4000 cells per condition). At the end of culture, the progeny from each well was plated in two CFU wells with MethoCult GF M3434 (StemCell technologies, Canada). The wells were imaged using StemVision (Stemcell technologies, Canada) and colonies were counted automatically using StemVision analyzer software. The average of the two wells was used to determine the colony count. Four independent experiments were carried out to obtain the final graph (Figure 3f).

## Supplementary Materials

The supplementary figures are available online at https://www.mdpi.com/1422-0067/21/17/6444/s1.
