# Supplementary material for: Mineral and Amino Acid Profiling of Different Hematopoietic Populations from the Mouse Bone Marrow
Source: Int J Mol Sci. 2020 Sep 3;21(17):6444. doi: 10.3390/ijms21176444 (PMC7504538; doi:10.3390/ijms21176444)
Supplement: Supplementary file 1 [file ijms-21-06444-s001.pdf]

Supplementary Figure 1

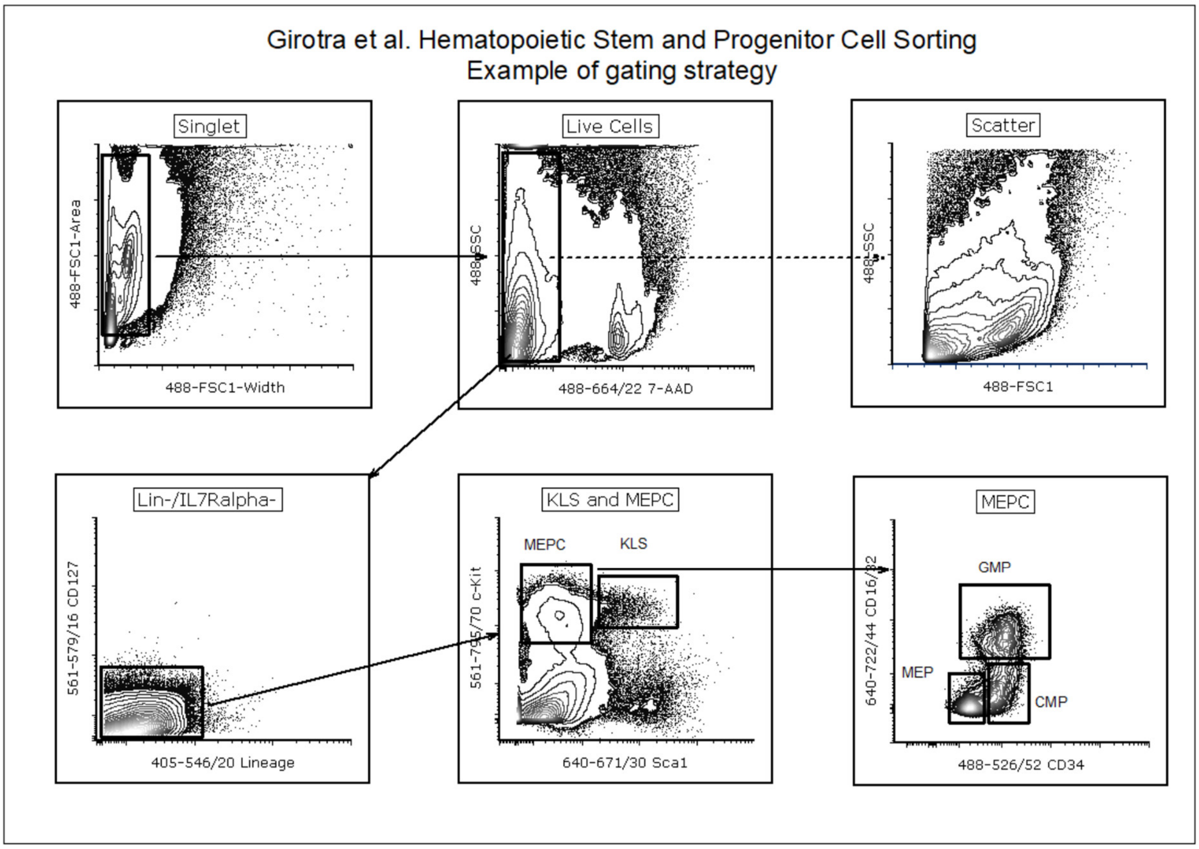

Supp Figure 1: Full Gating strategy to identify different HSPC populations from the mouse bonemarrow.

Supplementary Figure 2

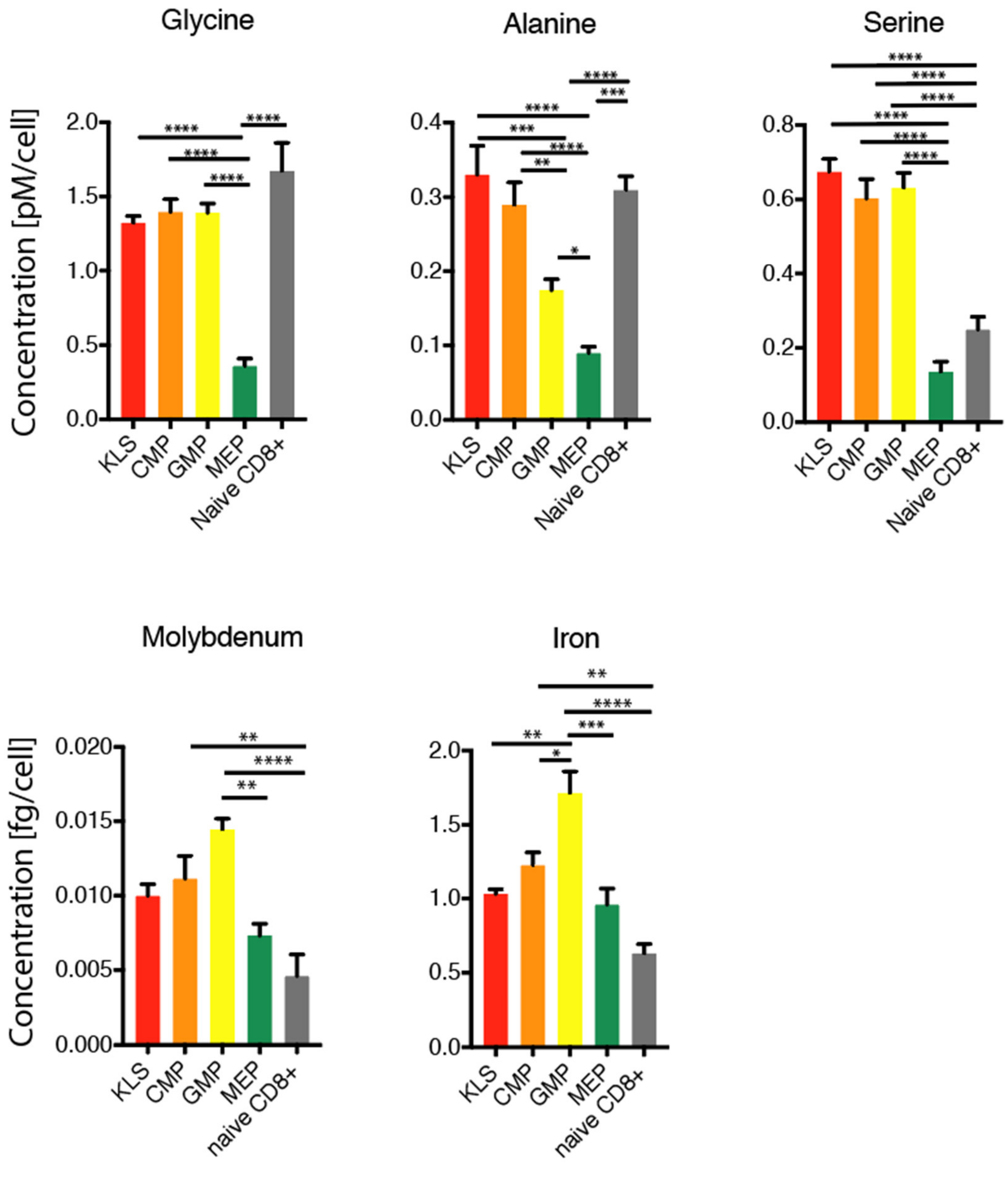

Supp Figure 2: Aminoacid and mineral profile comparison between HSPCs and CD8+ naive T cells. Different populations from the mouse BM and CD8+ naive T cells from mouse spleen were FACS sorted for amino acid and mineral analysis. KLS: Lin-cKit+Sca1+; CMP: Common Myeloid Progenitor; GMP: Granulocyte Macrophage Progenitor; MEP: Megakaryocyte Erythroid Progenitor; naïve CD8+: CD3+CD8+CD62L+CD44- . One-way Anova *p<0.05, **p<0.01, ***p<0.001, ****p<0.0001. (n=3)

Supplementary Figure 3

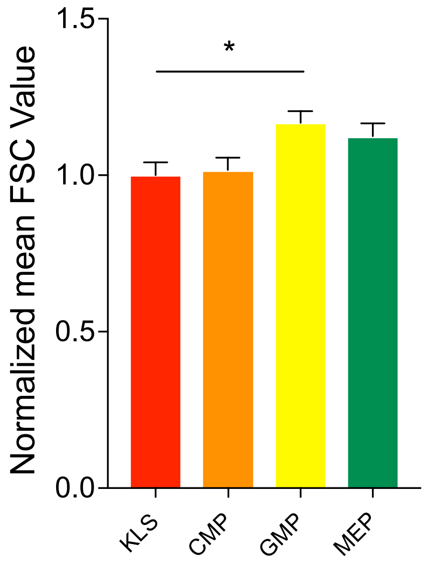

Supp Figure 3: FSC based cell size analysis of different bonemarrow HSPC populations.
One-way Anova *p<0.05, **p<0.01, ***p<0.001, ****p<0.0001. (n=6)
